# Design of a seniors and Alzheimer's disease caring service platform

**DOI:** 10.1186/s12911-021-01626-3

**Published:** 2021-11-16

**Authors:** Cheng-Wen Lee, Hsiu-Mang Chuang

**Affiliations:** College of Business, Chung Yuan Christian University, No. 200, Zhongbei Rd., Zhongli Dist., Taoyuan City, 320314 Taiwan, ROC

**Keywords:** Alzheimer, Location tracking, Fall detection, Internet of Things

## Abstract

**Background:**

To meet the needs of aging and dementia patients in Taiwan, this study designed a nursing system that includes communication, location tracking, and fall detection, and early warning services. The main purpose of this research is to provide timely services to the elderly and patients and hope to reduce the burden when the number of nursing staff decreases. This article is a remote disease care service platform with the Internet of Things (IoT) devices to monitor the location of the elderly and whether they have dropped warning alerts.

**Results:**

The device is connected to the patient's waist and chest, monitors the patient's movement and behavior, and transmits messages to the back-end system, and informs caregivers through mobile phone applications when unexpected or shocking events occur. The system can identify whether the patient has fallen, accidentally, or long-term inactivity. The device is equipped with sensors that enable it to monitor the patient's location and behavior data through Bluetooth and GPS technology. Finally, we proposed a basic model and an integrated model that will industrialize the system and is expected to play a role in a larger patient population.

**Conclusions:**

The system developed in this research has passed the Activities of Daily Living (ADL) test and verification, and is expected to provide appropriate safety care services for nursing homes and elderly residences.

## Background

### Motivation and purposes

Dementia is a disease phenomenon rather than normal aging. Many family members think that the patient is old-fashioned and stubborn. They think that this is the case when people are old, and therefore ignore the importance of seeking medical treatment. But in fact, he is already sick and should be treated. According to the International Dementia Association (ADI) Global Dementia Report 2019, it is estimated that there are more than 50 million people with dementia in the world, and it is expected to grow to 152 million by 2050. One person suffers from dementia every three seconds; the current cost of dementia is $1 trillion per year, and it is expected to double by 2030 [1].

According to the results of the dementia epidemiological survey conducted by the Taiwan Dementia Association commissioned by the Ministry of Health and Welfare (Republic of China), and the demographic data of the Ministry of the Interior of Taiwan at the end of December 108, it is estimated that there are 3,607,127 elderly people over 65 in Taiwan, of which There are 654,971 people with mild cognitive impairment (MCI), accounting for 18.16%; 280,783 people with dementia, accounting for 7.78% (including 114,336 people with very mild dementia, accounting for 3.17%, and 166,506 people with mild or more dementia, accounting for 4.62%) [2, 3]. In the classification of dementia, it can be roughly divided into two categories: degenerative and vascular, but patients sometimes have two or more causes, the most common is the coexistence of Alzheimer’s and vascular dementia (Also known as hybrid) [4, 5].

In recent years, there has been a lot of research on fall detection, and the main direction is to distinguish fall events from ADL (Activities of Daily Living) [6]. Because some ADLs (such as sitting down/from standing to lying down) have a high degree of similarity, it is necessary to collect user ADL data and data generated by volunteer simulations (signals such as acceleration, pressure), analyze the data, and perform Processing and classification. Fall detection systems are all made into wearable devices. At present, the research and development of devices such as smart clothes, foot sensors, and smartphones are continuing. However, related research points out that although the wrist-type fall detection device is convenient to use, the wrist is very unstable and it is very difficult to analyze the point where the center of gravity is shifted. If the fall detection device is placed on the wrist, the data stability will be greatly reduced. In contrast, the IoT waist-type fall detection device used in this research is located near the center of gravity of the body, and the data stability is high.

Therefore, due to the decline of the labor force due to the aging population in Taiwan, it is not easy to maintain the quality of care with limited human resources when the current care methods are highly dependent on manpower. More and more industries and research units are trying to use remote service mode-using IoT wearable devices with sensors to retrieve patient physiological information for analysis or emergencies. This study uses the above-mentioned technologies to construct related services to provide Alzheimer's disease caring service for the patients.

### Related works

The objective of this study is to combine theory and actual demand, and thereby provide user the truly effective help; in the simultaneity of discussing the related academic research, it is a must to consider the one whether there is practically difficulty when being used in the actual scenario, and attempt to solve all of these difficulties by trying feasible method. Firstly, we need the following technologies when analyzing such platform: falling detection, positioning, IoT equipment design, user demand (including user, user’s relative, career and operating management unit), where the positioning technology gets enough support by the existing scientific technology, so it is not discussed any more here.

Refer to the related studies of falling analysis papers, the famous research was the "Determining Activity Using a Triaxial Accelerometer" proposed by Mathie et al. [7]. it designed to interpret falling incident by combining accelerometer hardware and SVM (Signal Vector Magnitude) (refer Eq. 1), and use accelerometer to measures the acceleration value of three axes (x, y, z) to evaluate the signal vector magnitude threshold in order to determine whether people are falling.

1 $${\text{SVM}} = \sqrt {ax^{2} + ay^{2} + az^{2} }$$

Related studies continue to use the algorithm of SMA (Signal Magnitude Area) (refer Formula 2) and TA (Tilt Angle) (refer Formula 3) with accelerometer and gyroscope to analyze and improve the interpretation accuracy [8, 9].

2 $${\text{SMA}} = \frac{1}{t}\left[ {\mathop \smallint \limits_{0}^{t} |x\left( t \right){\text{d}}t + \mathop \smallint \limits_{0}^{t} |y\left( t \right){\text{d}}t + \mathop \smallint \limits_{0}^{t} |z\left( t \right){\text{d}}t + } \right]$$

3 $$\begin{aligned} &\rho = \tan^{ - 1} \left( {\frac{{\alpha_{x} }}{{\sqrt {\alpha_{y}^{2} + \alpha_{z}^{2} } }}} \right),\\&\varphi = \tan^{ - 1} \left( {\frac{{\alpha_{y} }}{{\sqrt {\alpha_{x}^{2} + \alpha_{z}^{2} } }}} \right),\\&\theta = \tan^{ - 1} \left( {\frac{{\sqrt {\alpha_{x}^{2} + \alpha_{y}^{2} } }}{{\alpha_{z} }}} \right) \end{aligned}$$

On the basis aforesaid, those studies in recent years have applied more methods to improve the interpretation accuracy, our explanation is made as follows [10–22]:

1. Higher ADL accuracy is acquired by using the noise of the input signal of Kalman Filter accelerometer and gyroscope.
2. Accuracy of accelerometer and gyroscope is improved, and volume and weight of wearable device are improved.
3. Place wearable device on the different area of human body, such as: wear on the hand, hung on the waist and put on the neck etc.
4. Acquire higher ADL accuracy by distinguishing the different falling styles with various thresholds: Fall forward, fall backward, slow fall, trip and unexplained ways to cause a fall.
5. Interpret whether fall incident happens by using camera and motion detection.
6. Enter the information of accelerometer and gyroscope into the neural network model to analyze and acquire higher ADL accuracy.
7. Enter the information of accelerometer and gyroscope into the deep learning model to analyze and acquire higher ADL accuracy.

However, the following issues are found in the related studies upon the interviews with many relatives, nursing home managers and caregivers:

1. A higher falling accuracy of old people can be interpreted by camera, but it infringes the right of privacy, so cameras are prohibited in old people’s home and public area in many countries or regions.
2. Related ADL studies are based on accelerometer and gyroscope, and considering the fall risk of the old people, all tests are done with the assistance of young people; for old people and young people, there is huge difference in their fall force and style. Fabio Bagalà and Clemens Beckerh made practical verification before. In the actual living environment of old people, the accuracy in the technology verified by ADL is reduced too much.
3. For the wearable device, such as watch, it may detect wrongly since both hands act violently in the usual time.
4. According to the investigation of Taiwan ministry of health and welfare (8), 44.4% old people’s fall occurred at their own home, where 26.6% occurred in bathroom or toilet, 22.91% in living room and 13.7% in bedroom. According to the actual investigation data of old people’s home, the place at which old people may very possibly fall down at home is the bedside (when going to bed or getting out of bed and bathroom (taking a bath), the percentage of falling down at such place is 70%; however generally, old people may not wear the wearable device in such two periods; if old people falls down outside the room, the most important thing is to let his/her relative know the situation and location of old people as soon as possible, so it is more important to achieve a design combining warning, telephone conversation and positioning alarm than the independent fall alarm; but there is few study on discussing the system mechanism like this.
5. Even though neural network model or deep learning model may generate accurate information, wearable device usually uses the thresholds those are fixed constants, and many studies focus on the interpretation basis served by the fixed thresholds recorded by the APP program of cell phone. However practically, many old people and Alzheimer's patients are not used to wear the smart cell phone, so the similar methods can’t be widely applied; moreover, it is easier to cause higher false alarm rate for different old people and Alzheimer's patients when using fixed thresholds.
6. Due to the limited working staff in old people’s home, higher false alarm rate in the wearable device may cause big puzzles to caregivers. However, the related supporting measures are still not available. When an old people fall down, it may give rise to the caring dispute, and his/her relative may also encounter the same puzzles.
7. For many old people’s homes, the manner of location tracking has been adopted to judge and interpret the issue whether old people fall down or not, the commonest one is people halting or loitering detection technique.

By gathering all issues aforesaid for analysis, we adopted two methods, namely positioning and falling alarm in our work to carry out our study, and solved the problem of relative and carer practically by simplifying systematic operation process. Moreover, we adopted the manner of interview for our verification, and found that it was beneficial for carer and relatives.

## Methods

This platform mainly proposes two functions.

A. Refer to the suggestions of the employees in the cooperative nursing homes and related reference documents to combine theory and practice.
B. The platform is integrated with the developed residential system, and IoT equipment is used to meet actual needs.

### Front-end IoT device

**Fig. 1:**
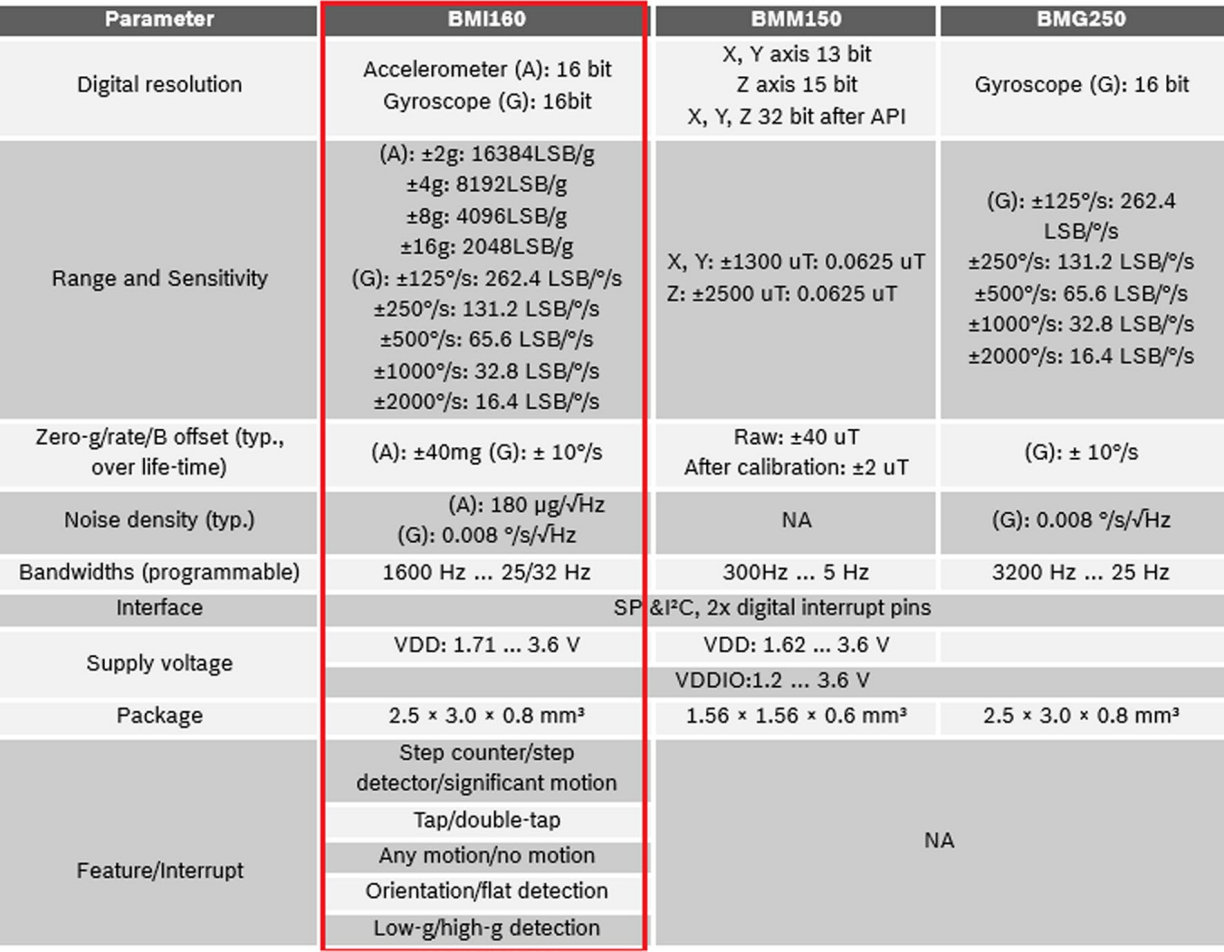
BMI160 IMU specification

For the Front-end IoT device designed by this study, it mainly contains three components: IMU (inertial measurement unit, with the accelerometer and gyroscope contained) as needed by fall detection, GPS positioning chip, MCU (Microcontroller) and telecommunication transmission subassembly. Among which, IMU is the BMI160 adopting Bosch Sensortec, its specification shown in Fig. 1 [23].

**Fig. 2:**
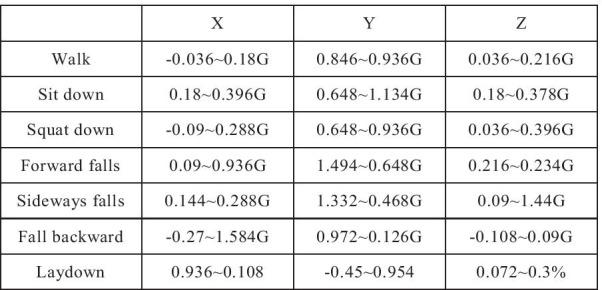
BMI160 IMU specification

Currently, commercial IMUs provide fixed thresholds for human inertial measurement actions (refer Fig. 2). but such thresholds cannot be applied to everyone, (For example, young people and old people have different action thresholds). For this part, we will discuss in next section.

**Fig. 3:**
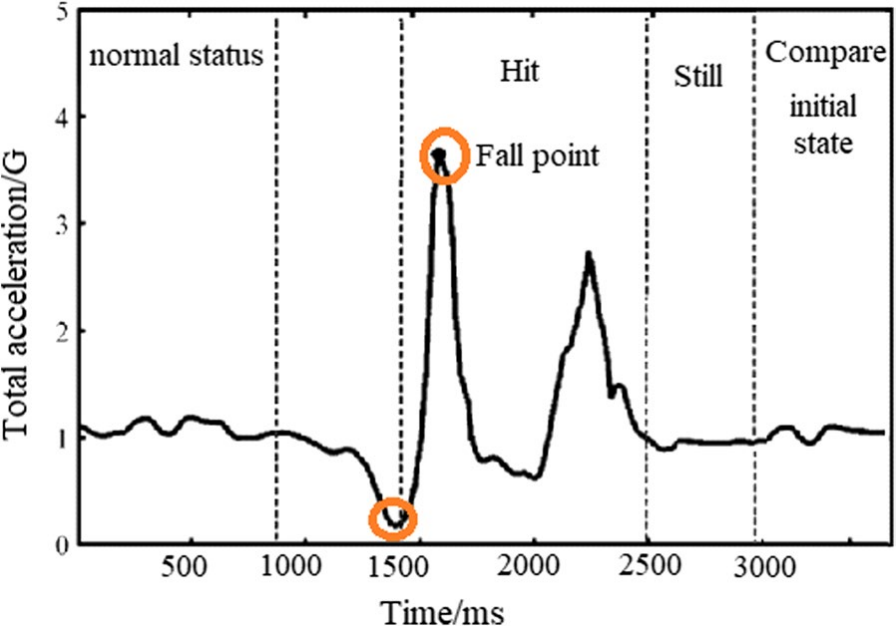
The acceleration variation model of human falling

The fall behavior is the phenomenon that the body of a person loses its balance suddenly and falls down accidently. In the state of falling, the force suffered by person may change along with the accelerated speed of body movement. The state of falling contains four stages (refer Fig. 3):

- (1) weight loss: at the beginning of fall, body may lose the weight assuredly. When the acceleration magnitude changes suddenly, the tilting angle of body may rapidly increase; for the general fall, the resultant acceleration generated by the weight loss may be less than 1 g (in the general condition, the resultant acceleration therefrom may be larger than 1 g). Therefore, it can be used as the first judgment basis for the state of falling. (2) Hit: when falling down, the body of a person may hit against the ground or other article, it can generate the reverse change in the curve of acceleration. So, it can be used as the second judgment basis for the state of falling. (3) Motionless: after falling down, namely after hitting against ground or other articles, the body of a person may keep motionless for a while. It can be reflected by the still movement in the curve of acceleration. (4) Comparable initial state: after falling down, the state of body may be different from its initial state, this is the judgment basis of the one whether the falling result is severe or not. Referring to the picture: in this continuous behavior, the corresponding thresholds can be used to evaluate.

The IoT device used in this system has the following specification.

- It has telecommunication and call functions, and can provide emergency call services (establish a whitelist to control dial-in and dial-out).
- GPS location and network transaction, with the function of connecting to wireless networks, it can automatically switch and upload data to cloud indoors and outdoors, and save the customers telecommunication network costs.
- Standby for more than 72 h under normal use, and a reminder message will be sent out when the battery is low; those who meet the requirements of assistive devices can get subsidies for use.
- Built-in accelerometer and gyroscope, provide datasets for use in fall status evaluation.
- With radio function and remote parameter update and shutdown functions.
- It has all the hardware and firmware design specifications to become a personal portable care device.

In our study, the datasets (the x, y, and z accelerometer linear acceleration data, and the ρ, φ, θ gyroscopic angular velocity data) from the IMU of smart box are not totally submitted to the back office for analysis, instead, the records in two minutes when alarm is triggered (despite of true alarm or false alarm) will be submitted for analysis. For the true alarm or the false alarm, caregivers of old people’s home or relative of old people may confirm and then annotate, which will be used for the back-office analysis. This method is the result from the continuous discussion in the verification process with the nursing home managers.

### Server-side adaptive falling detection methods

Based on the related works, this study adopted the LSTMs (Long Short-Term Memory cells) technique for fall detection, the major reason is the practical consideration, resource conservation (telecommunication network fee, electric power of equipment’s battery, computing ability of front/rear-end device), aiming at providing appropriate solutions to wearers, relatives, management staff of organization and caregivers.

This study also adopts IMU (BMI160), the sensor signals (accelerometer and gyroscope) were pre-processed by applying noise filters and then sampled in fixed-width sliding windows. The sensor acceleration signal, which has gravitational and body motion components. All related data will be entered into the LSTMs model of server for analysis.

Comparing with the traditional method, by using an RNN (Recurrent Neural Networks) of LSTMs, the data can be directly fed to the neural network, which can achieve an easier training; moreover, it can avoid what is called the vanishing gradient problem [24–26].

This article uses Google's neat Deep Learning library, Tensor Flow, as explained in this article, an RNN takes (x, y, z, ρ, φ, θ) dataset as input vectors to process them. The output for the model will be a six-element vector containing the probability of a given window belonging to each of the six activity types thresholds (Falling forward Falling back right, falling right, falling left, Falling back left and Trip over).

**Fig. 4:**
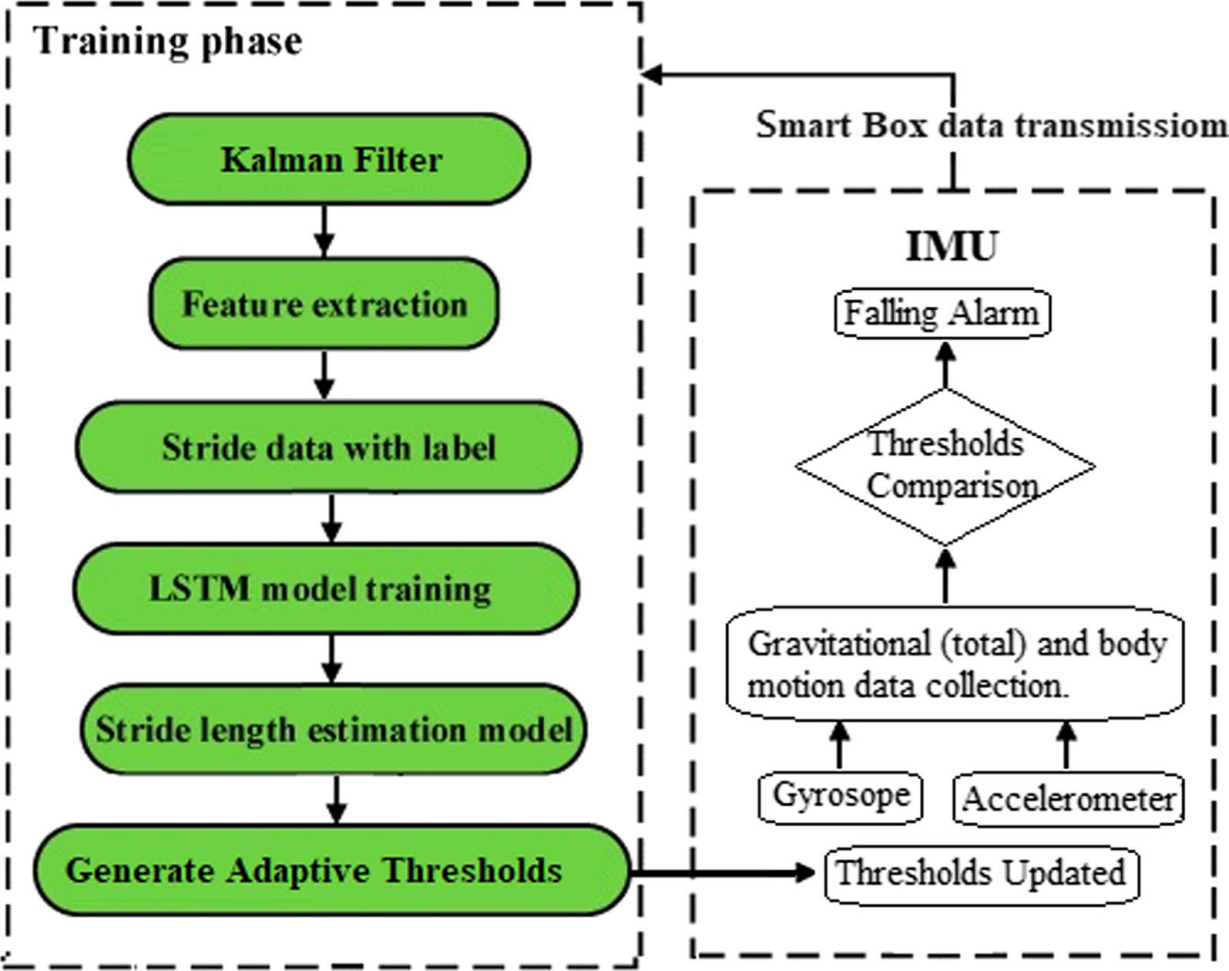
Dataset training flow

We define the model as having a single LSTM hidden layer, and connect a dropout layer after the hidden layer in order to reduce the model's overfitting to the training data. Next, a dense fully connected layer is used to interpret the features extracted by the LSTM hidden layer, and the result is poured to the final output layer. All optimized thresholds can be updated to the front-end smart box so as to provide the practical fall detection and judgment basis (refer Fig. 4).

### Care information management subsystem and mobile phone APP

**Fig. 5:**
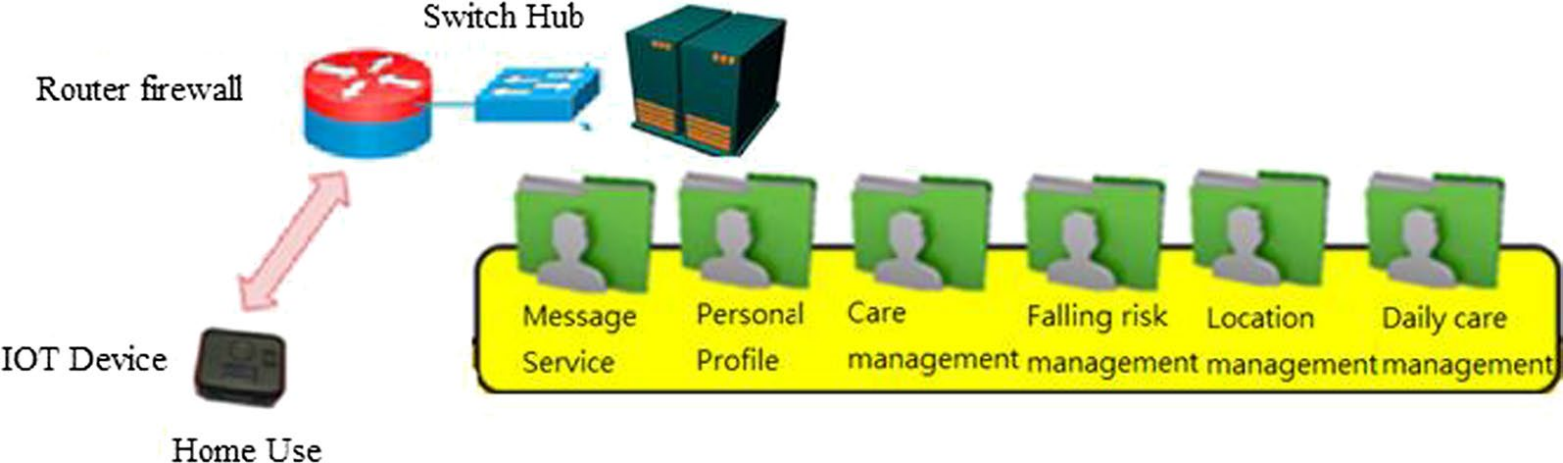
Network and host configuration architecture—basic mode (home)

Receive information from the message management system, and record front-end mobile phone APP and smart box related information. It consists of six components: A. Message service B. Personal profile C. Care management D. Falling risk management F. Location management E. Daily care management. The future is based on maintenance. Institutional needs mediate the resident system (refer Fig. 5).

**Fig. 6:**
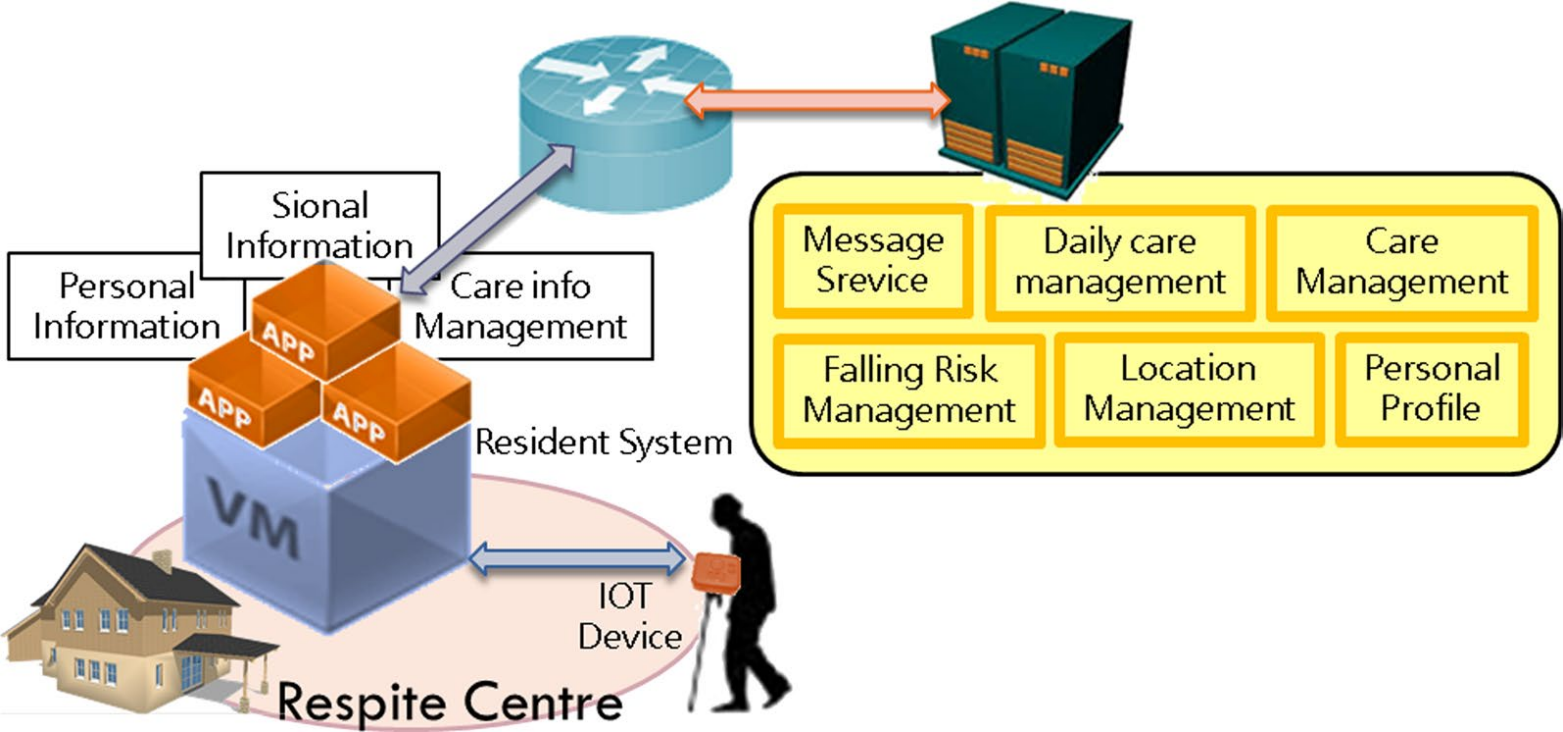
Network and host configuration architecture—extended mode (nursing home)

- Basic model: When there are few users at the beginning of the period, considering the operation and management costs, the relevant subsystems are constructed on the same entity host.
- Integration model: When working with pension institutions and other organizations, the related subsystems can be constructed on virtual machines in different locations through the integration model (refer Fig. 6). Information on the maintenance organization will be managed by the long-term care central platform. The subsystem is dispatched to the message management subsystem of the maintenance organization. The wisdom box information was also sent to the long-term care central platform.

Therefore, due to the need for rapid establishment of information architecture among the above-mentioned modes, planning and system setting can be used to achieve demand planning that can be modularized, scalable, and highly loaded.

The information delivery between device and server of this portfolio is explained as follows:

- Collect and transmit information related to the message management subsystem. The above plans, except for the front-end mobile phone APP, are constructed on the host of the back-end computer room and are planned in accordance with the requirements of modularity and expandability.
- In the operation of measuring value uploading, measuring numerical value inquiring, etc., one of the member ID number or member simple identification data may be selected as the primary key (hereinafter referred to as member identification data), and it is not necessary to transmit the identity at the same time. Certificate number and personal identification information of the member.
- Measurement data upload: The information transmission box will then encode the membership identification data and physiological measurement data in JSON format. After the encoding is completed, it will be uploaded to the security platform.

Archiving of measurement data: The platform will use the identification information of the member as the key value of the measurement data received and archive it into the member's record.

Care of the information management subsystem planning related data structure and Web API interface, refer to related data planning, in order to integrate or interface with other systems in the future. The related data structure of care information management subsystem planning and Web API interface, refer to relevant data planning so that it can be integrated or connected with other systems in the future.

## Experiments

This system includes the following two parts: location tracking and fall detection:

### Location tracking

**Fig. 7:**
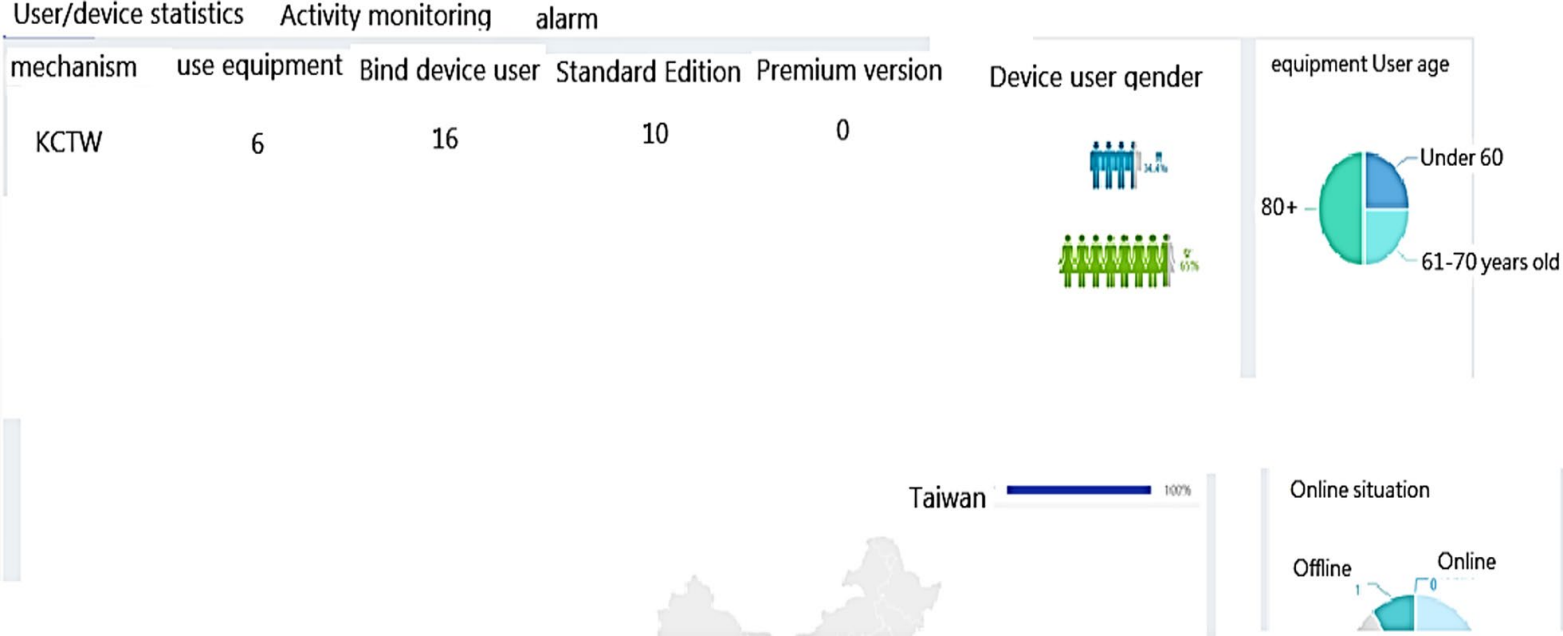
Personal daily activity management (nursing home)

**Fig. 8:**
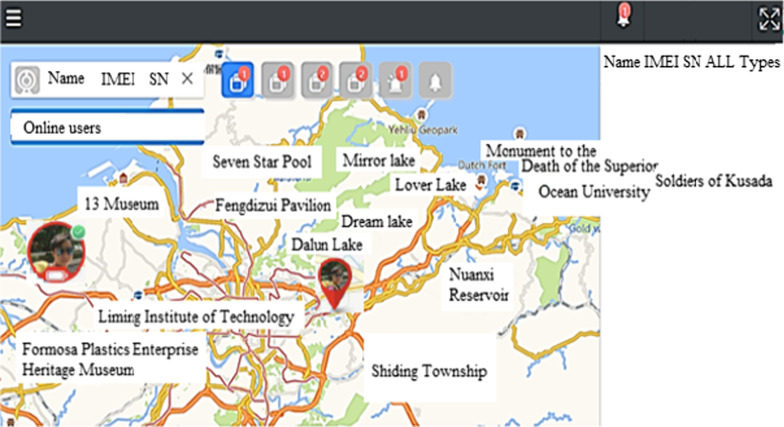
Real-time tracking monitoring

**Fig. 9:**
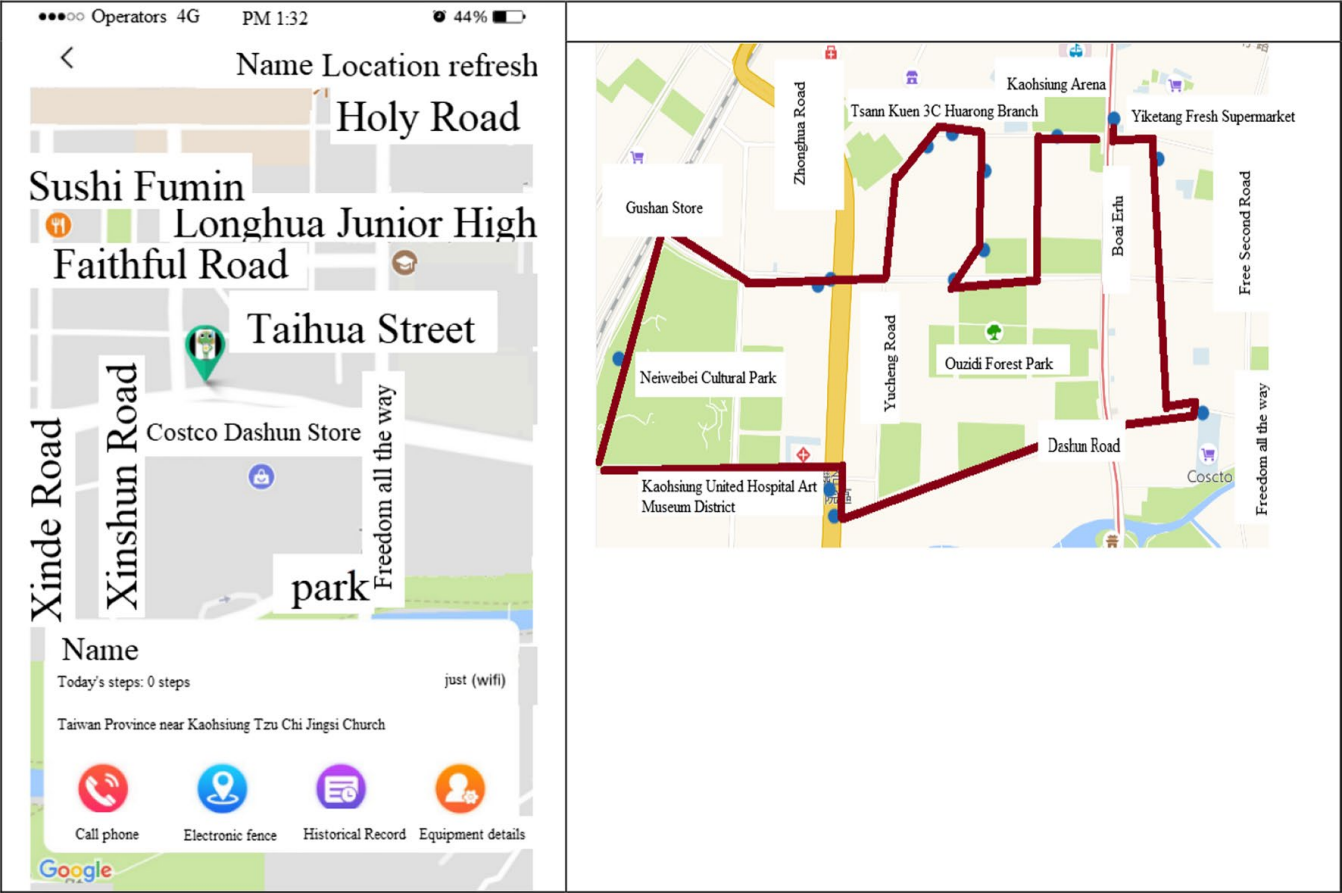
Real-time location tracking monitoring

**Fig. 10:**
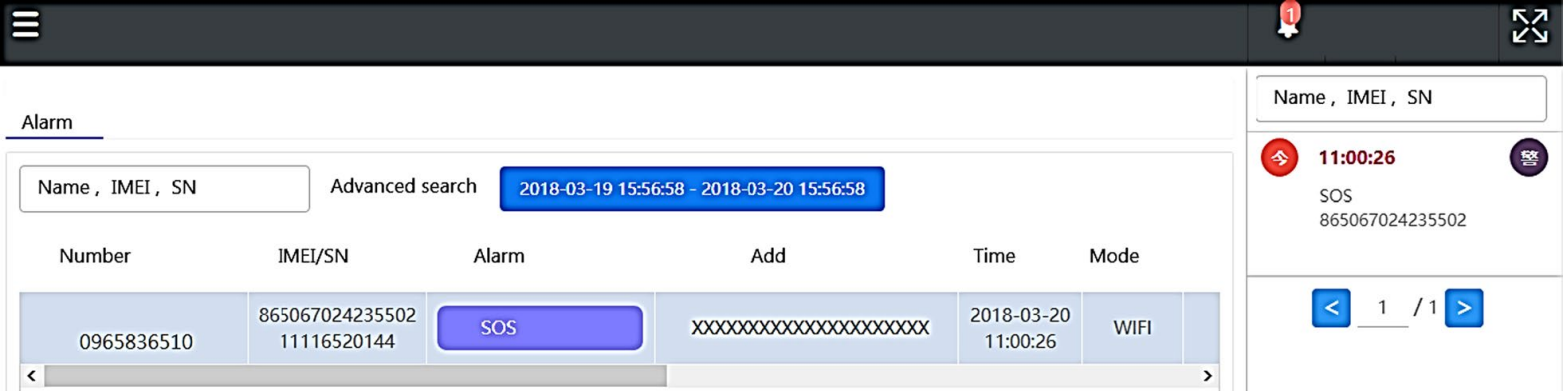
Wearer message processing

**Fig. 11:**
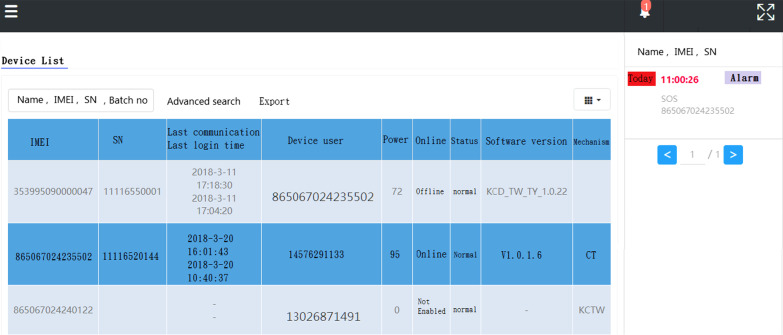
Equipment management

1. Data statistics: according to the user's data, the wearer's organization, sex, age, region …etc. (refer Fig. 7).
2. Real-time monitoring (for institutional management): smart box reports relevant information (based on the user's name, IMEI code, and SN code, GPS data) to the back-end location management subsystem, the subsystem provides a personnel location interface combines with Google map service to caregiver and family members, they can track any time through the computer or the mobile app. The interface used by the nursing home stuffs can also track the location information of all wearers at the same time (refer Fig. 8).
3. Tracks of movement (institutional management or family members): You can use the date range to query the wearer's footprint, trajectory, and heat map (in conjunction with the number of movement steps, calories, rhythm, trajectory analysis) (refer Fig. 9).
4. Message processing (institutional management): institutions can search for information on abnormalities such as alerts, SOS activation, home, away from home, access to electronic fences, etc. for their occupant wearers (refer Fig. 10) [16].
5. Equipment management (institution management or family use): Information about the smart box bound to an APP account can be queried, including information about the wearer's information, smart box power, whether it is online, and the organization or family member (refer Fig. 11).

Verification: Long-term Care Facilities (partner-institutional nursing home).

Stability: Normal test/sample number of various functions of nursing information management system. Table 1 is the daily location of the wearer of the Internet of Things device monitored by the caregiver, and Table 2 is the relevant statistical information of the caregiver and the elderly inquired by the back-end manager A.

**Table 1:** ADL location and tracking test process

Item times	Measures	Observation	Results
A1	Daily home Stay- Wearer1	Steps 1, 2, 3, 4	P
A2	Daily home Stay- Wearer2	Steps 1, 2, 3, 4	P
A3	Daily nursing home location- Wearer3	Steps 1, 2, 3, 4	P
A4	Daily nursing home location- Wearer4	Steps 1, 2, 3, 4	P
A5	Daily nursing home location- Wearer5	Steps 1, 2, 3, 4	P
A6	Location tracking in Kaohsiung city- Wearer6	Steps 1, 2, 3, 4	P
A7	Location tracking in Taipei city- Wearer7	Steps 1, 2, 3, 4	P
A8	Location tracking from Taipei city to New Taipei City- Wearer8	Steps 1, 2, 3, 4	P
A9	Location tracking in Taoyuan city- Wearer9	Steps 1, 2, 3, 4	P
A10	Location tracking from Taipei city to Taoyuan City- Wearer10	Steps 1, 2, 3, 4	P

*Test data used for employees of both companies, non-institutional data

Test result: stability = 100% (pass/sample) (refer Table 2)

**Table 2:**
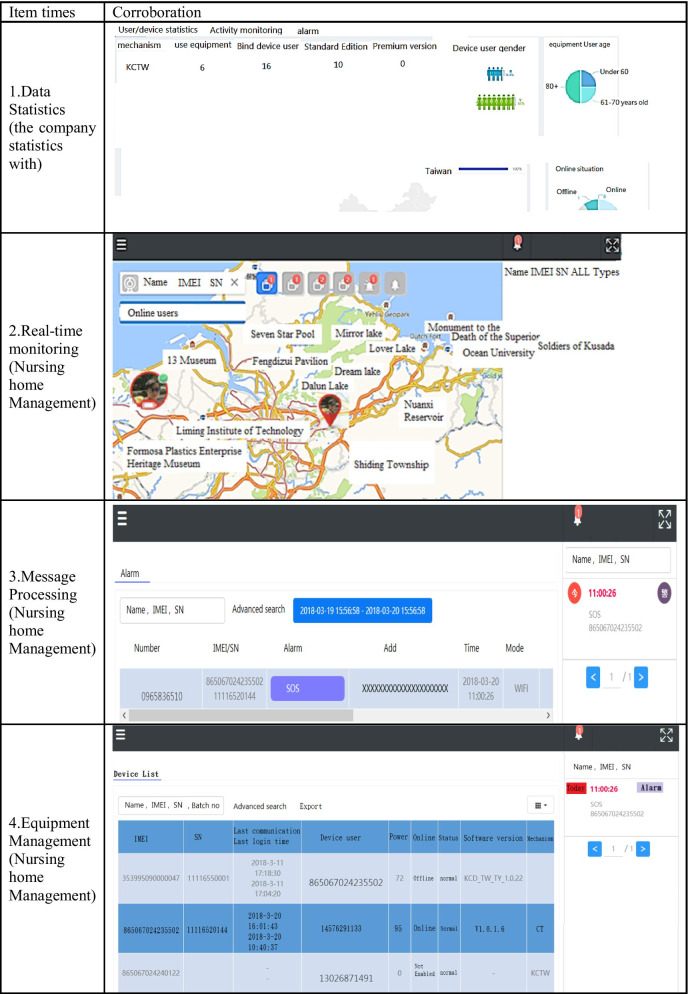
ADL caregiver management process

ADL test process (Number of samples: 10):

1. Start the program.
2. Test function:
  - Statistics (for vendor A statistics).
  - Real-time monitoring (for institutional management).
  - Message processing (for institutional management).
  - Equipment Management (Organization Management).
3. Observe that the reaction is correct.
4. Assessment result: Pass [Failed]/Fai [Failed].

KPI requirements: stability = 100% (pass/sample).

### Fall detection ADL

1. The IoT device used in this system use accelerometers and gyroscopes for detection; accelerometers measure the Cartesian coordinate (x, y, z) (including the result of gravitational acceleration), and gyroscopes measure the spherical coordinates (ρ, φ, θ)-the angular velocity of rotation. Use changes in different body postures (for example, changes in body acceleration and direction) to determine if a person has fallen (refer Table 3).
2. ADL experiments: We simulate 6 situations as follows: a. Falling forward b. Falling left c. Falling right d. Falling back left e. Fall down right back f. Trip over. The KPI requirements for fall state assessment:
  True Positive (TP): is the number of falls detected in the fall sample (true alarm)
  True Negative (TN): is the number of samples detected as not falling (not alarmed)
  False Positive (FP): is the number of falls detected in samples that have not fallen (false alarm)
  False Negative (FN): is the number of fall samples detected as not falling (not alarmed)

**Table 3:** ADL falling detection test process

Simulate situations	Simulate situations
1.Falling forward	2. Falling left
3.Falling right	4.Falling back left
5.Falling back right	6.Trip over

1 ~ 6 tests: 50 ADL tests for each item, a total of 300 tests.

Real test results get data: TP = 145, TN = 136, FP = 9, FN = 10.

- ACC (Accuracy Rate) = (TP + TN) / (TP + TN + FP + FN) = 93.7%
- FPR (False Positive Rate) = FP / (FP + TN) = 6.2%
- FNR (False Negative Rate) = FN / (TP + FN) = 6.5%

During the cooperation period of this project and nursing home, there were three true falls in the care recipients, the accuracy rate is 100%.

### Wearing rules

#### Nursing homes certification

**Fig. 12:**
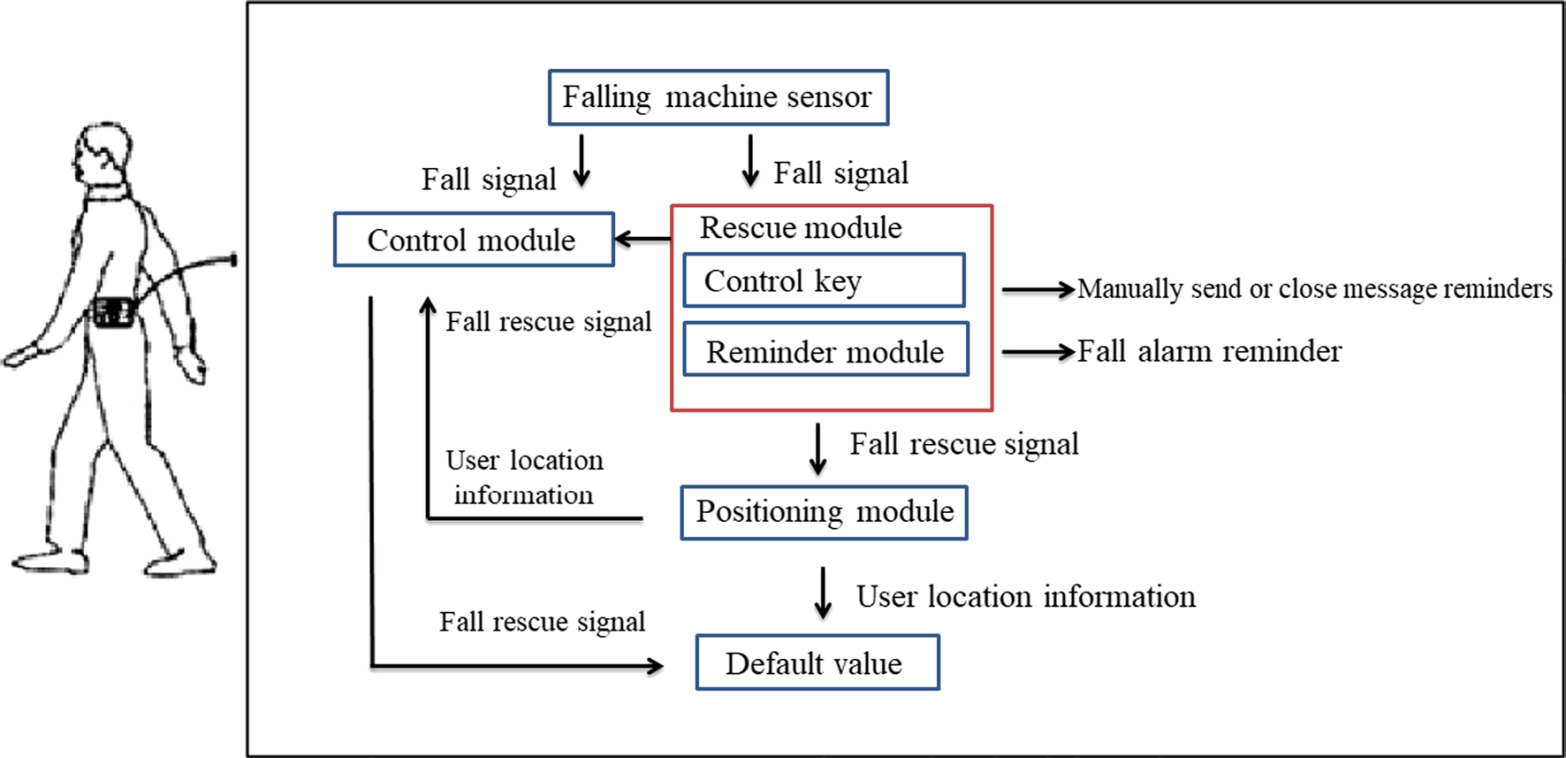
Operation flow chart

The subsystem provides with Google map service to caregiver and family members, they can track any time through the computer or the mobile app. The interface used by the nursing home stuffs can also track the location information of all wearers at the same time (Fig. 12).

**Fig. 13:**
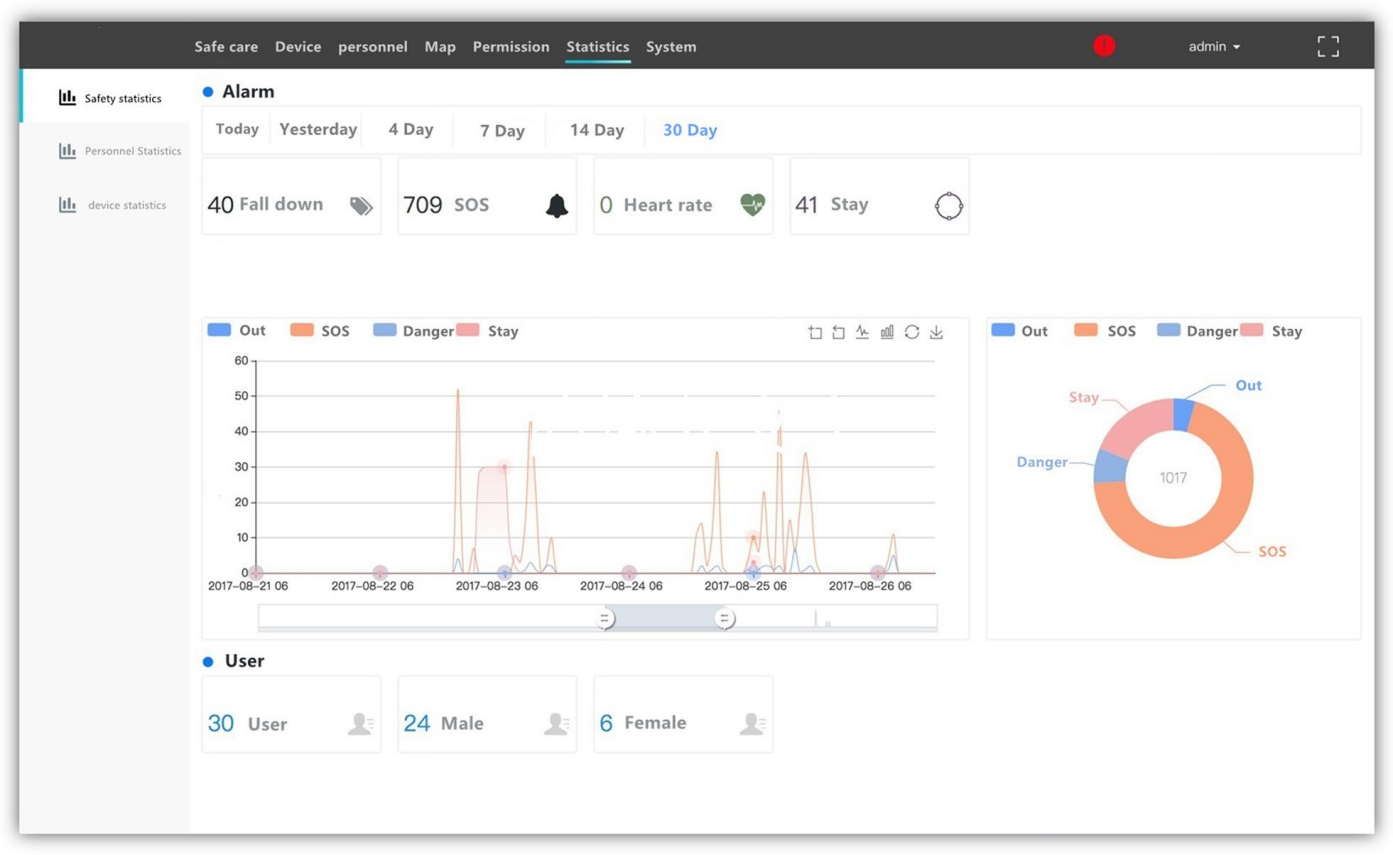
Background control management

The back- end of the subsystem is connected to the data collection host and the analysis database. Through the WEB interface, the management staff of nursing homes, inpatient service staff and residents' families can analyze and use the collected data. We also provide a simple back-end control platform in Fig. 13.

## Results

The subsystem provides with Google map service to caregiver and family members, they can track any time through the computer or the mobile app.

The interface used can also track the location information of all wearers at the same time.

## Discussions

In this study, we interviewed caregivers of those organizations providing cooperation to our experiment and relatives of old people (including 25 caregivers and 15 relatives); those caregivers achieved 85% satisfaction for the use of the system (17 felt satisfactory and 3 felt unsatisfactory), those relatives achieved 93% satisfaction for the use of the system (14 felt satisfactory and 1 felt unsatisfactory). Among them, 8 caregivers had the experience in nursing the old people wearing smart watch, 6 relatives had the experience in nursing the old people wearing smart watch at home. For the interview on the issue whether smart watch is helpful for their nursing operation, caregivers achieved the satisfaction of 25%, relatives achieved the satisfaction of 17%. There are two main reasons:

1. Smart watch is complicated to operate for the old people who really needs nursing, so they dislike to wear it.
2. The smart watch usually must match with smart cell phone, the complicacy in the combined use of both of them and the need of charging smart equipment frequently may give rise to huge troubles to old people.
3. The alarm style is difficult to personalize (including the alarm sound of watch and the instructions how to remove false alarm), and there are no integral back-office supporting measures (positioning/staying information, whether the two-way telephone call can be achieved when the alarm triggers, whether the emergency button can trigger an alarm etc.) for supporting caregivers and relatives.
4. If the false alarm is triggered at higher rate, the difficulty in judging the real alarm will increase and which will cause overmuch mental stress to caregivers and relatives (especially the relatives).

Relative to the issues aforesaid, the platform in this study can provide sound solutions and different from the concept of the research in this work is established on the development software platform of “mobile care carrier”, aiming at providing the safe care service for the seniors and the populations with dementia and integrating the miniature smart box and the software, upon the SWOT analysis.

## Conclusions

With the continuous improvement of Internet of Things technology and information technology, in recent years, the alert SMS service of wearable devices combined with information has gradually attracted attention. Taiwan has entered an aging society, which has changed the age structure of the population, and has also promoted the focus of medical services from emergency treatment to gradually Focus on chronic disease treatment and safety care.

The platform includes the functions of care and physiological measurement, which is designed to ensure that the operator can master the information of the monitoring site. For example, in the family or pension institutions, when the manpower is weak or late at night, the alarm is sent by telephone voice or alarm message to inform the family or the staff on duty to deal with the emergency immediately, so as to effectively prevent the accident from expanding and reduce the loss.

Remote fall protection platform falls detection is one of the innovative applications of the application of capital communication technology in preventive health care, medical care and care and other related services. The application of remote health care services to the health management of chronic patients the platform is responsible for improving the effect of health care through monitoring and even the self-management awareness of the user.

## Data Availability

All data generated or analysed during this study are included in this published article.

## Abbreviations

IoT: Internet of Things
GPS: Global positioning system
ADI: Alzheimer's Disease International
MCI: Mild cognitive impairment
ADL: Activities of daily living
SMA: Signal magnitude area
TA: Tilt angle
IMU: Inertial measurement unit
MCU: Microcontroller
RNN: Recurrent neural networks
LSTM: Long short-term memory
IMEI: International mobile equipment identity
SN: Serial number
TP: True positive
TN: True negative
FP: False positive
FN: False negative
ACC: Accuracy rate
FPR: False positive rate
FNR: False negative rate
